# Premedical special master’s programs increase USMLE STEP1 scores and improve residency placements

**DOI:** 10.1371/journal.pone.0188036

**Published:** 2017-11-30

**Authors:** Bryan Johnson, Matthew Flemer, Sadik Khuder, Nitin Puri

**Affiliations:** 1 Dept. of Physiology and Pharmacology, College of Medicine and Life Sciences, The University of Toledo, Toledo, Ohio, United States of America; 2 Dept. of Medicine, College of Medicine and Life Sciences, The University of Toledo, Toledo, Ohio, United States of America; 3 Dept. of Medical Education, Joan C. Edwards College of Medicine, Marshall University, Huntington, West Virginia, United States of America; Charles P. Darby Children's Research Institute, 173 Ashley Avenue, Charleston, SC 29425, UNITED STATES

## Abstract

The effectiveness of Special Master’s Programs (SMPs) in benefiting a potential medical student’s career beyond admission into an MD-program is largely unknown. This study aims to evaluate the role of SMPs, if any, in affecting the performance and outcomes of students during their medical school career. This study analyzed anonymous surveys of students and residents from the University of Toledo. The data analysis is used to evaluate a student’s academic performance before, during and after medical school. Measured metrics included: MCAT Scores, undergraduate GPA, USMLE STEP 1 scores, participation in research, number of research publications, and residency placement. Of 500 people surveyed 164 medical students or residents responded. Based on their responses, the respondents were divided into traditional (non-SMP) and SMP groups. As anticipated, MCAT scores (SMP: 29.82 vs. traditional 31.10) are significantly (p<0.05) different between the two groups. Interestingly, there is no significant difference in USMLE STEP 1 scores (SMP: 232.7 vs. traditional: 233.8) and when normalized relative to MCAT scores, USMLE STEP 1 scores for SMP-students are significantly (p<0.05) higher than their traditional counterparts (p<0.05). Additionally, SMP-students did not outperform the traditional students with regards to research publications. But, they did demonstrate a significant (p<0.05) proclivity towards surgical residencies when compared to the traditional students. Overall, our results highlight that SMPs potentiate USMLE STEP 1 performance and competitive residency-placements for its students.

## Introduction

A Special Master’s Program (SMP) is a one to three-year program that is designed to assist a college graduate’s transition into a post-secondary professional school, such as medicine or law [1]. In the last eight years, the master’s programs designed for those trying to gain admission into medical school have seen a surge in popularity, resulting in the establishment of over one hundred and thirty-five different programs nationwide—a forty-five percent increase since 2009 [2]. These programs offer students an opportunity to engage with graduate level material and demonstrate their ability to master some of the complex topics they will encounter as they progress through medical school. The prevailing belief is that a student's performance in such a program can be easily measured and serve as a great tool for admissions boards in determining an applicant’s qualifications as a potential medical student [1,3]. However, only a handful of studies have investigated the impact of these master’s programs on academic outcomes in medical school.

To date, research analyzing SMPs has narrowly focused on institutional specific goals and/or the success of underrepresented minority groups. In this regard, Epps, et al., demonstrated that the SMP at Meharry Medical College was successful in positioning its students to be competitive with their medical school peers with regards to USMLE STEP 1 and 2 pass rates, as well as graduation rates [4]. Additionally, Giordani et al., suggested that SMPs were able to successfully prepare individuals with lower MCAT and GPAs to be competitive in their first year of medical school, with a specific focus on underrepresented groups [5]. While these studies establish a baseline for SMP efficacy, they are not all-encompassing. Evidence is currently lacking whether SMPs are successful for students regardless of socioeconomic background, in addition to whether these programs have an impact on student’s USMLE STEP 1 scores or residency placements.

The investigation of these unknowns is important as not only can the SMP programs take up to 3 years to complete, but they are also expensive. While tuition rates vary, it can cost up to $30,000 for a one-year program [3]. Given the length and cost of these programs, as well as the gaps in the current body of research, we sought to observe the degree to which SMPs are able to influence the student’s academic performance throughout their medical school career. To this end, we analyzed survey-data from medical students and residents currently at UTCOM. This is one of the first studies investigating the outcomes of students who participated in various SMPs by analyzing metrics that have direct correlation with medical school success including MCAT score, USMLE Step 1 score, participation in medical research, and residency placement.

## Methods

This study was reviewed and approved by the Social, Behavioral and Educational Institutional Review Board of the University of Toledo, College of Medicine and Life Sciences. This study was deemed IRB exempt (protocol number is 0000202178).

This study analyzed anonymous surveys of students and residents from the University of Toledo. We analyzed the performance of medical students and residents with regards to their undergraduate GPA, MCAT and USMLE STEP 1 scores as well as research publications, comparing SMP-students (respondents that completed an SMP) and traditional students (respondents that did not complete an SMP).

Since participating medical-students and/or residents may have completed an SMP from institutions beside UT, we inquired about their respective SMP characteristics (length, research involvement, etc.), so that more nuanced comparisons could be made. Additionally, we asked residents to list their specialty so that we can see if and how SMP participation affects residency placements. The survey-data was then analyzed to draw specific conclusions.

### Survey design

A fifteen-question anonymous survey (S1 File), using Survey Monkey, was sent out to UT residents and medical students who had taken USMLE STEP 1. A portion of their MCAT score was reported within a narrow range and was subsequently averaged for statistical analysis.

### Participants

Medical students and residents were targeted for this study, and subsequently divided into two groups based on their completion of an SMP (SMP-students) or not (traditional students). Majority of the SMP-students completed their SMP at the University of Toledo, however, the cohort also included SMP-students from Georgetown University, University of Cincinnati College of Medicine, Wayne State University and Loyola University Chicago. All data is completely anonymous.

### Statistical analysis

**Fig 1:**
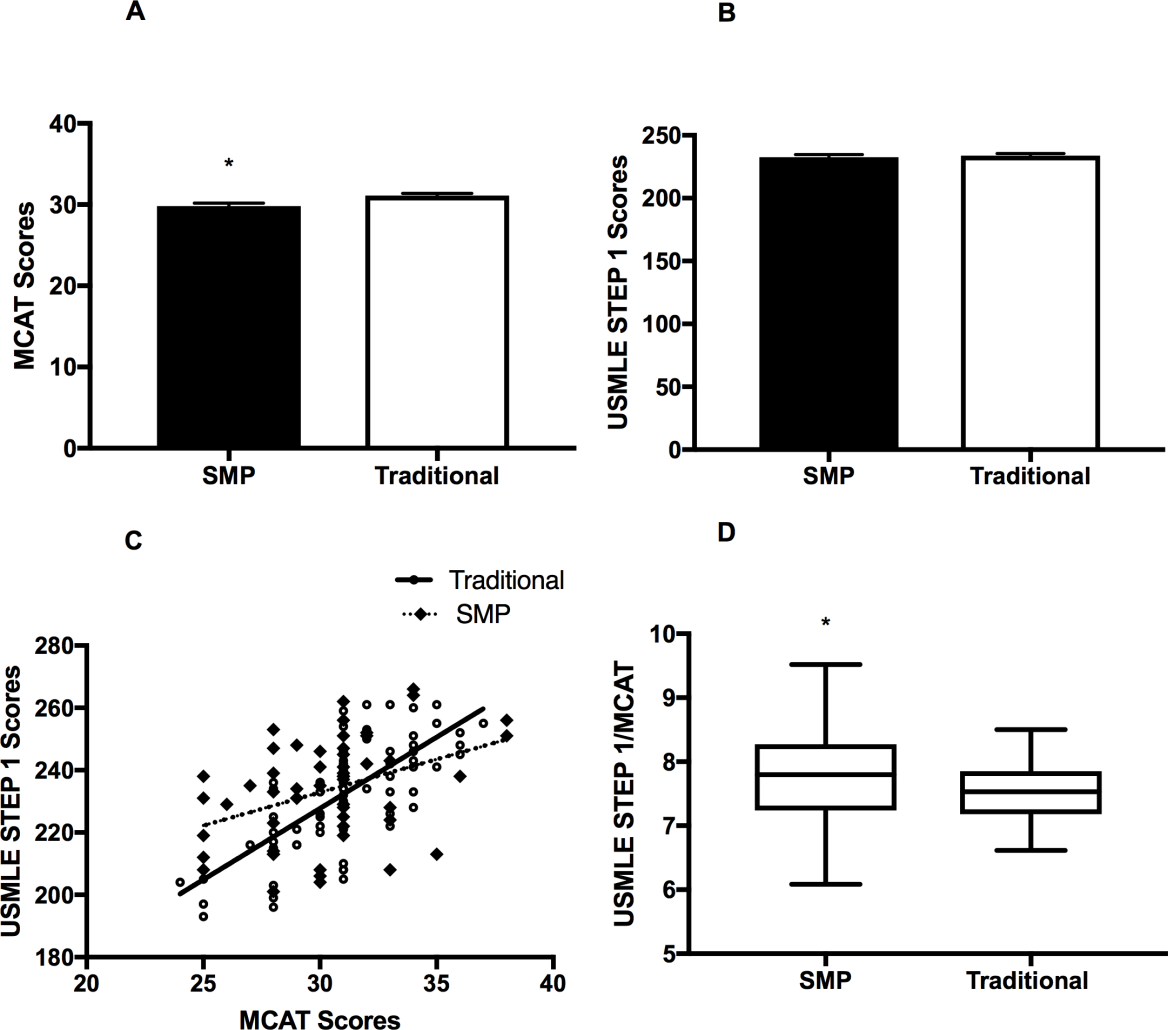
A. Comparative Analysis of MCAT Scores between SMP and Traditional Students. Unpaired T-Test shows that MCAT scores for incoming Traditional Students (mean = 31.1(0.2784 SEM), n = 87) are significantly higher (p<0.01) than incoming SMP Students (mean = 29.9 (0.3578), n = 56). B. Comparison of USMLE STEP 1 Scores between SMP and Traditional Students. Unpaired T Test shows that USMLE STEP 1 scores of SMP (mean = 232.7 (2.091 SEM), n = 54) and Traditional Students (mean = 233.8 (1.616 SEM), n = 82) are not significantly different. C. MCAT vs. USMLE STEP 1 Plot and Trends of SMP and Traditional Students. Linear regression analysis of MCAT and STEP 1 scores in SMP Students (slope = 2.126, r^2^ = 0.1473, n = 54) and Traditional Students (slope = 4.571, r^2^ = 0.5138, n = 77) shows that the trends for the two groups are significantly (p<0.01) different. D. Comparison of USMLE STEP 1 Scores, normalized to MCATS, between SMP and Traditional Students. Unpaired T test shows that MCAT-normalized, USMLE STEP 1 scores of SMP (mean = 7.77 (0.10 SEM), n = 54) and Traditional Students (mean = 7.51 (0.05 SEM), n = 77) are significantly (*p<0.02) different.

**Fig 2:**
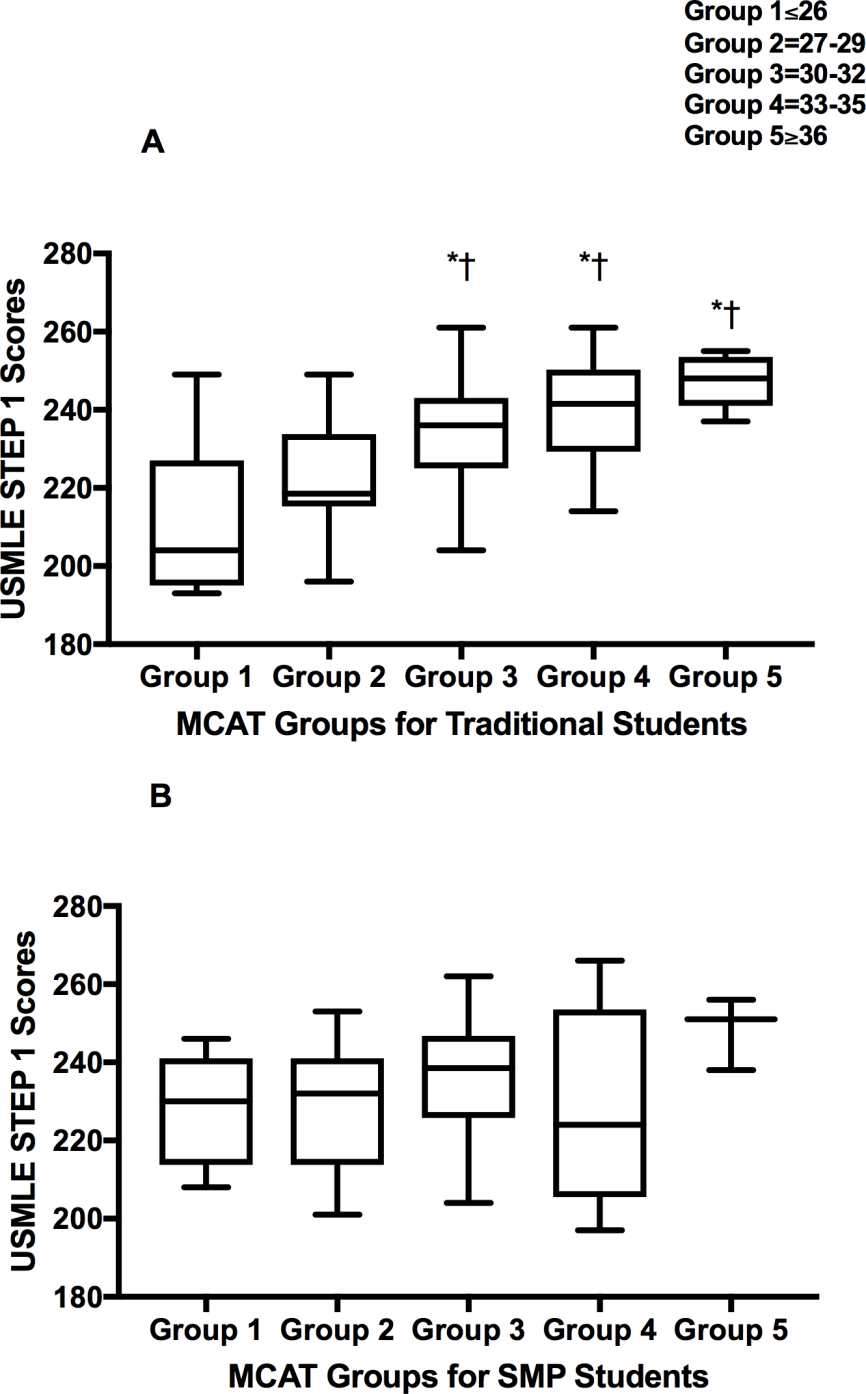
**USMLE STEP 1 Scores Based on MCAT Groupings for Traditional (A) Students and SMP-students (B).** Students were categorized into 5 groups based on MCAT scores (Group 1 = 26 and below; Group 2 = 27–29; Group 3 = 30–32; Group 4 = 33–35; Group 5 = 36 and above) and then further divided into SMP and Traditional students and comparative analysis was performed using One-way ANOVA with Tukey Kramer Post-hoc analysis. * = p<0.05 vs. traditional group 1; ✝ = p<0.05vs. traditional group 2.

Data collected from our surveys were organized in Microsoft Excel spreadsheets and subsequently analyzed using GraphPad Prism 7 software. To limit variability, values between the 5^th^ and 95^th^ percentile are included in the analysis for data shown in Figs 1 and 2. Specific details of the statistical methods involved are included in figure legends.

## Results

In total, the aforementioned survey was sent out to 500 potential respondents, of which 164 responded. For this analysis, we excluded 8 respondents who did not attend medical school in the US. Of the 156 remaining, 95 of the respondents were traditional students, 61 of were SMP-students. We mined the survey-data to detect any differences, or lack thereof, between the SMP and traditional students’ performance before, during, and after medical school. To accomplish this we focused our examination on survey-data pertaining to undergraduate GPA, MCAT scores, USMLE STEP 1 scores, research, and residency programs.

### MCAT and USMLE STEP 1

Standardized exams including MCAT and USMLE STEP 1 minimize room for subjectivity and provide for a uniform metric of performance for both SMP and traditional students. As shown in Fig 1A traditional students scored significantly (p<0.05) higher than SMP-students with regards to MCAT. However, as shown in Fig 1B, no significant difference in average USMLE STEP 1 scores is observed between the two groups. SMP-students had means of 29.8 and 232.6 on the MCAT and USMLE STEP 1, respectively, whereas traditional students showed means of 31.1 and 233.8, respectively.

With the unexpected similarities in USMLE STEP 1 averages we next sought to examine the correlation between MCAT and USMLE STEP 1 for both groups. As anticipated, traditional students displayed a positive correlation (R^2^ = 0.51) between these two exams–students that scored higher on the MCAT most likely scored higher on USMLE STEP 1, and vice versa. Importantly, however, SMP-students demonstrated a significantly (p<0.01) less positive correlation between MCAT and STEP 1 scores (R^2^ = 0.15) (Fig 1C).

While the previously mentioned findings were significant, the impact of the SMP remained unclear. To investigate such an association, we normalized the USMLE STEP 1 scores relative to MCAT scores for each student. Normalized USMLE STEP 1 scores for the SMP students is significantly (p<0.05) higher as compared to the traditional students (Fig 1D). This reveals a potentially positive impact of the SMP on the students’ STEP 1 performance.

Next, we organized traditional and SMP-students into 5 groups based on their MCAT performance (Group 1 = 26 and below; Group 2 = 27–29; Group 3 = 30–32; Group 4 = 33–35; Group 5 = 36 and above) and compared each group’s average USMLE STEP 1 score with that of every other group. Notably, for the traditional students, MCAT groups 3, 4, and 5 have significantly (p<0.05) higher USMLE STEP 1 scores as compared to both groups 1 and 2 (Fig 2A). These results provide additional evidence of a positive correlation between USMLE STEP 1 and MCAT among traditional students.

Importantly, there is no significant difference between the USMLE STEP 1 scores of any of the MCAT-groups of SMP students (Fig 2B). This data reaffirms our observation that USMLE STEP 1 scores for the SMP-students do not adhere to the strong correlative nature of their MCAT scores as seen in traditional students; all groups perform comparably to their peers.

### GPA and USMLE STEP 1

**Fig 3:**
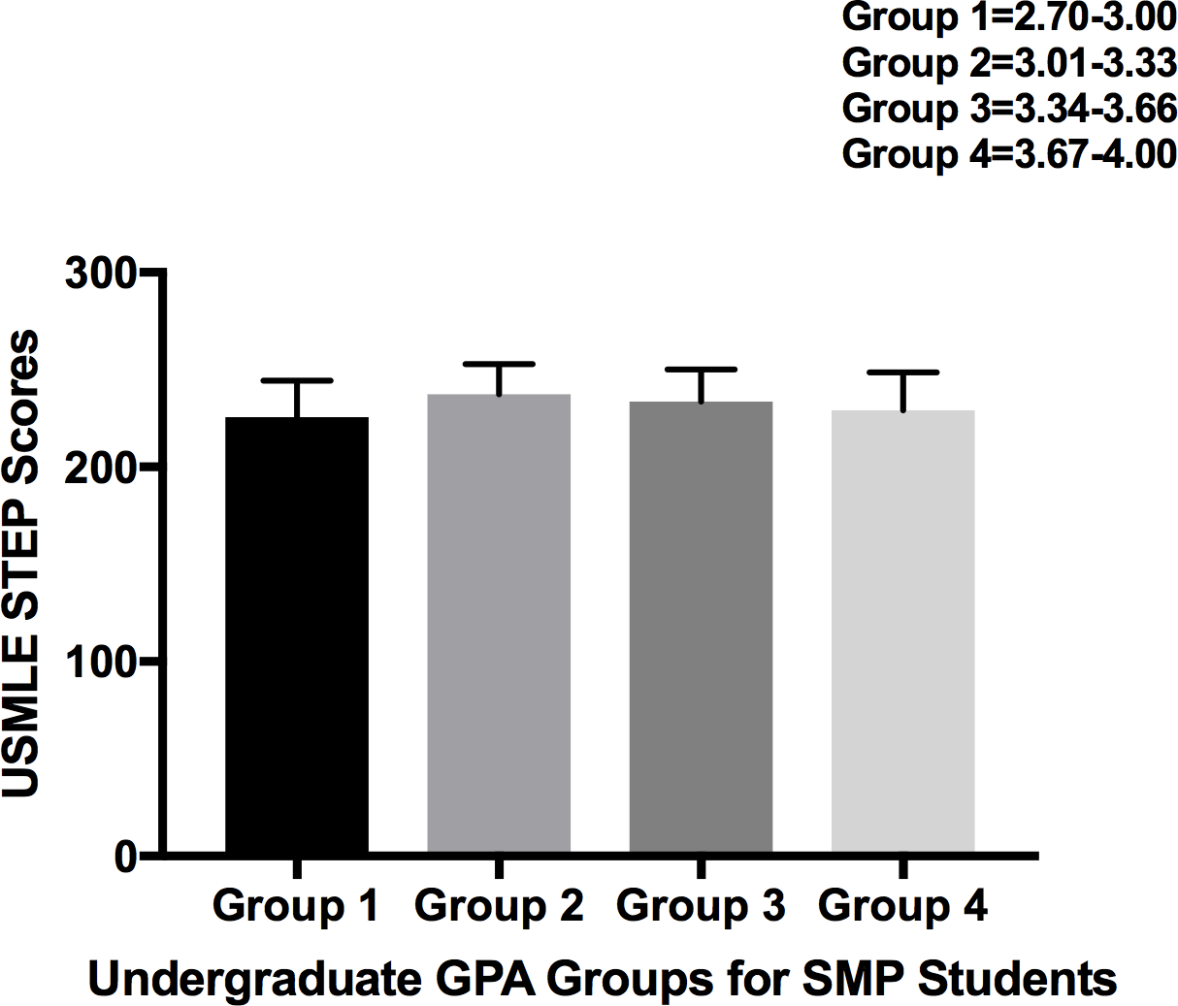
Comparative analysis of USMLE STEP 1 scores relative to undergraduate GPAs of SMP students. SMP students are categorized into 5 groups based on undergraduate GPA scores (Group 1 = 2.70–3.00, Group 2 = 3.00–3.33, Group 3 = 3.34–3.66, Group 4 = 3.67–4.00) and comparative analysis was performed using One-way ANOVA with Tukey Kramer Post-hoc analysis.

In order to ascertain the predictive value of an undergraduate GPA, if any, we organized SMP-students into four groups based on GPA (Group 1 = 2.70–3.00, Group 2 = 3.00–3.33, Group 3 = 3.34–3.66, Group 4 = 3.67–4.00) and analyzed the USMLE STEP 1 scores of those groups. These results failed to reveal any significant differences among the GPA groups with regards to their USMLE STEP 1 performance in both traditional or SMP students (Fig 3).

### Research activity

**Fig 4:**
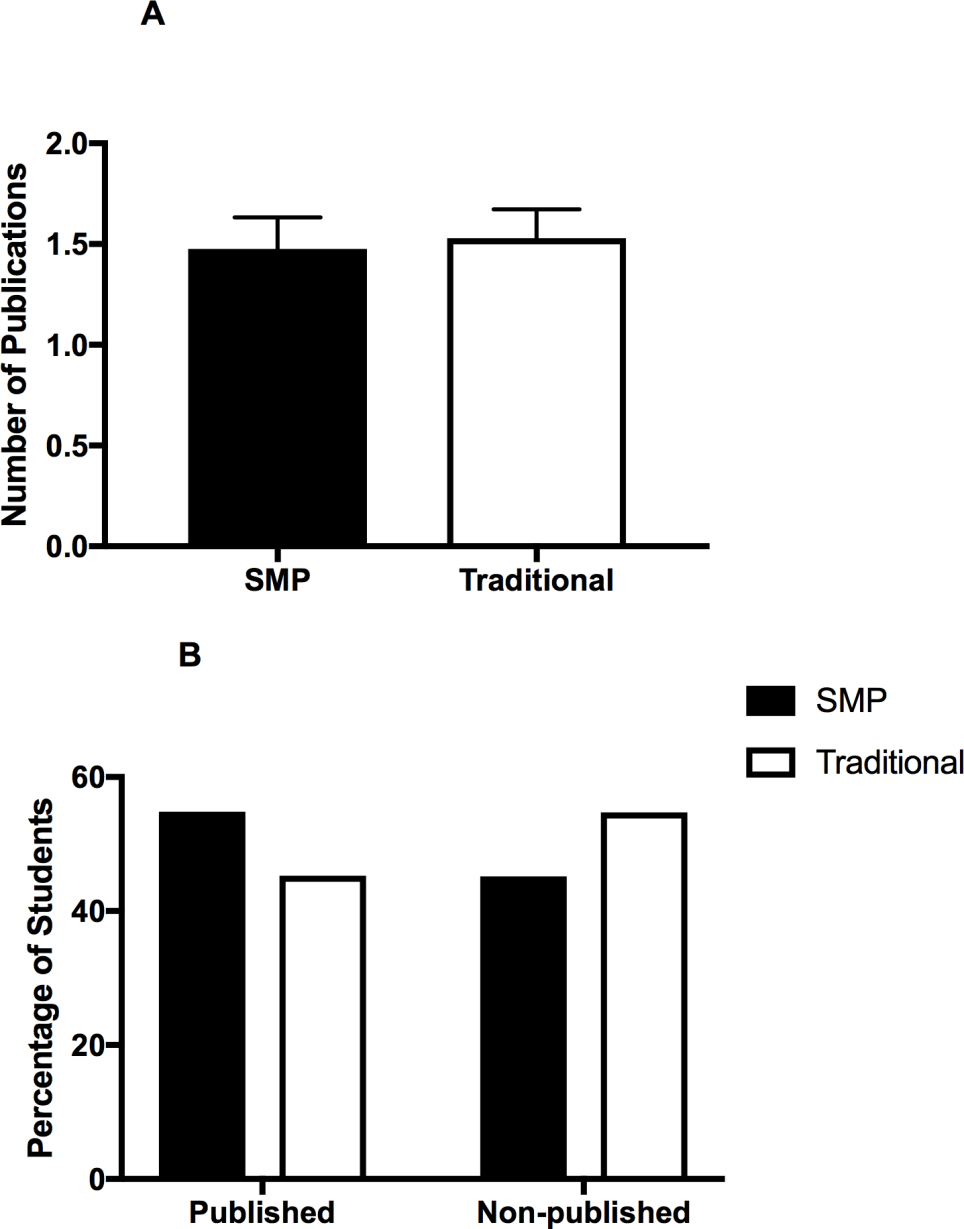
A. Comparative Analysis of Number of Publications between SMP and Traditional Students. Comparative analysis using an unpaired t test shows no significant difference in the number of publications produced by SMP (mean = 1.48 (0.16 SEM), n = 42) vs. Traditional Students (mean = 1.53 (0.14 SEM), n = 51). B. Comparative Analysis of Percentage of Published and Non-Published SMP and Traditional Students. Chi Square analysis of students with at least one publication shows no significant difference between SMP (54.84%, n = 62) and Traditional Students (45.26%, n = 95).

As it is becoming an increasingly important metric in establishing competitiveness for residency applicants, we looked into research involvement and publications by students while in medical school. Although more SMP students tended to participate in research, the data did not achieve statistical significance. There is no significant difference in the percentage of SMP (68%) and Traditional (54%) students that participate in research (p = 0.08) (figure not shown), nor the average number of publications between SMP (1.4 publications per student) and traditional (1.45 publications per student) students (Fig 4A). Additionally, when examining the composition of published and non-published students within each group, there is no significant difference between SMP (54.84%) and traditional (45.26%) students (Fig 4B).

### Residency programs

**Fig 5:**
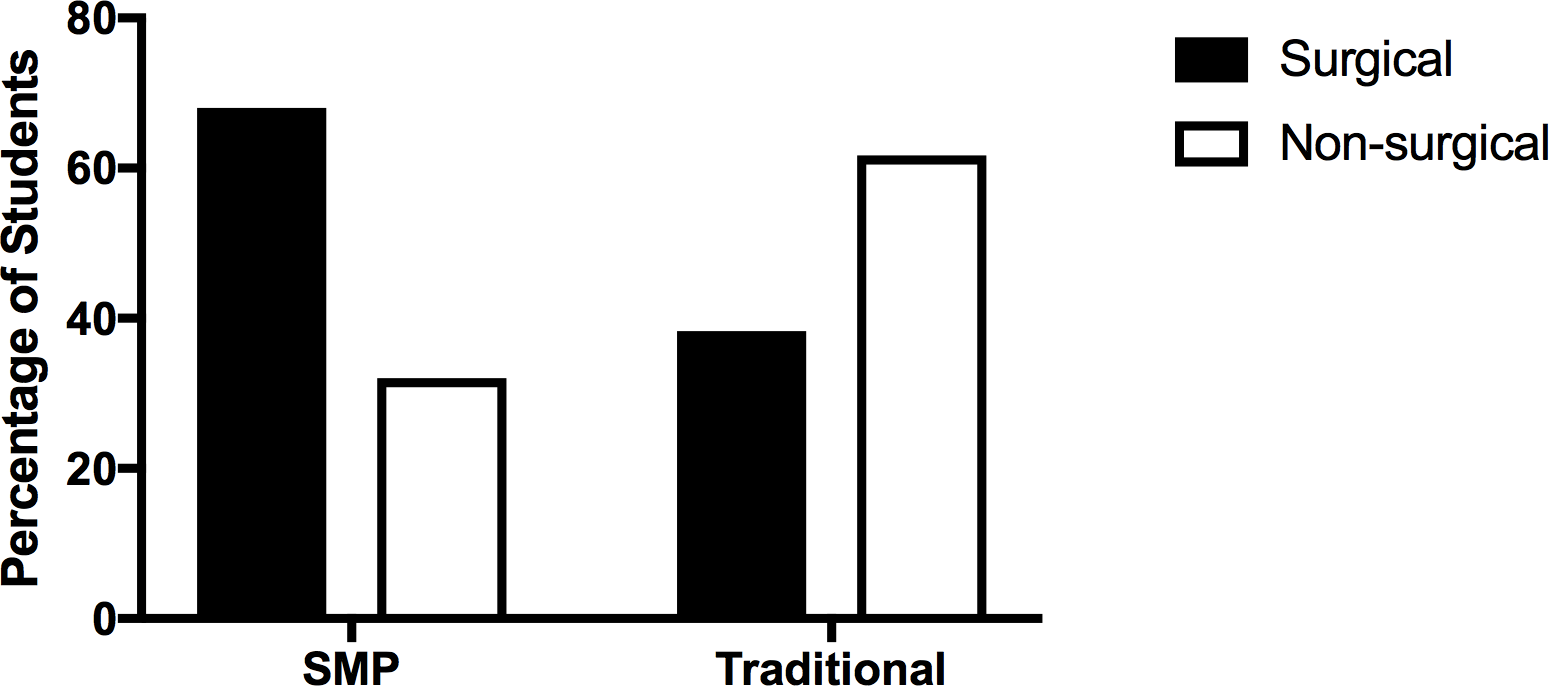
Comparative analysis of residency distribution between SMP and traditional students. 72 of the total 130 respondents reported being in a residency program. 25 were SMP-students and 47 were traditional students. Residency programs were classified into two categories: surgical subspecialties and non-surgical subspecialties. The surgical subspecialty category is comprised of orthopedic surgery, plastic surgery, general surgery, urology, obstetrics and gynecology, anesthesiology, and emergency medicine. The non-surgical subspecialty category is comprised of family medicine, pediatrics, pathology, internal medicine, psychiatry, radiology, neurology, and physical medicine and rehabilitation. Of the SMP respondents 17 (68%) were accepted into surgical subspecialties whereas 8 (32%) were accepted into non-surgical subspecialties. Of the traditional respondents 18 (38.3%) were accepted into surgical subspecialties and 29 (61.7%) were accepted into non-surgical subspecialties. Chi-squared analysis revealed this difference in distribution to be significant (p<0.02).

Competitive residency placement is a desired outcome of a successful undergraduate medical career. It is, in turn, critically dependent on key variables, including student’s USMLE Step 1 score and research participation. As such, we compared residency placements for our students finishing medical school, with or without participation in a special master’s program. As shown in Fig 5, the ratio of SMP students getting accepted into surgical subspecialties is significantly higher than the ratio of traditional students. While we recognize that some non-surgical subspecialties are as competitive as surgical subspecialties, it is noteworthy that SMP students are either applying or being accepted into the competitive surgical residency programs at a higher rate compared to traditional students.

## Discussion

SMPs are an invaluable tool for students seeking to gain admission into increasingly competitive medical school programs. However, the value of these master’s programs beyond medical school admission remains unclear. On the one hand, proponents may believe that an additional year of graduate level studies could assist SMP-students in medical schools by preparing them beyond the scope of their undergraduate studies [1,3]. Conversely, it could be argued that given the rising rate of burnout among physicians, the additional year of studies could actually hamper an SMP-student’s chances of success [6,7]. Thus, this study examines the long-term value of an SMP through the lens of several metrics that are currently used to evaluate the competency and preparedness of medical students for residency. Overall, our results defend our hypothesis that an SMP may hold its value for a student beyond admission into medical school.

In this regard, our first key finding is that when normalized relative to MCAT scores, USMLE STEP 1 performance for SMP-students was significantly higher than their traditional counterparts (Fig 1D). This is noteworthy given the lack of significant difference between the reported USMLE STEP 1 scores of SMP and traditional students (Fig 1B). Our initial observation indicated that when it comes to standardized testing in medical school, the intervening act of an SMP may not provide any additional benefit. However, normalizing the MCAT and USMLE STEP 1 scores uncovers a potential hidden benefit of the SMPs, where SMP-students on average preform higher than their traditional counterparts on USMLE STEP 1 relative to their MCAT. For example, using normalized values from Fig 1D, a traditional student who scored a 30 on their MCAT is likely to score a 225.3 on the USMLE Step 1, however, that same student is likely to improve their score to a 233.0 if they completed an SMP. Stated differently, for the mentioned student, an SMP could be the difference between being in the ~40^th^ percentile or the ~50^th^ percentile of medical students that take the USMLE STEP 1 exam [8].

Saguil et al. has previously observed an association between medical students’ performance on MCAT and USMLE STEP 1 [9]. Our results support these previously published findings with regards to traditional students, as seen in Fig 1C [9,10]. However, the data gathered among SMP-students fails to follow this strong correlation. The trend for SMP-students is of minimal correlation between MCAT and USMLE STEP 1 (r^2^ of 0.15). This notion is further supported by the fact that when comparisons are made within the group for traditional students, lower MCAT predicates lower USMLE STEP 1 scores (Fig 2A). Not only does the SMP break this trend, but also it is important to note that the lowest MCAT-group for SMP students perform at par with the higher MCAT-groups for the traditional students (Figs 1A and 2B). This suggests the inherent value of an SMP program in helping students outperform their predicted USMLE STEP 1. Thus, an SMP effectively generates a level playing field for all students regardless of their MCAT score. Rephrased, students who perform poorly on the MCAT and complete an SMP are not seemingly predestined to perform poorly on the USMLE STEP 1 exam, as are their traditional peers.

Given the minimally-correlative relationship between MCAT scores and USMLE STEP 1 performance in SMP-students, we sought to examine GPA as a potential USMLE STEP 1 performance-predictor as it could be indicative of underlying qualities such as effort and determination. Statistical analysis of differences in GPA scores for SMP students failed to show any changes in USMLE STEP 1 performance (Fig 3), thus negating the previously mentioned notion. This reinforces our conclusion that for students completing an SMP the correlative nature of their undergraduate credentials may not be accurate.

No single explanation can reconcile the fact that SMP-students are not bound by the predictive value of their MCATs or undergraduate GPAs. One possibility could be that an additional year of graduate level studies provides students with an opportunity to consolidate the groundwork for some of the challenging material that they will encounter throughout medical school. Our survey found that most of the respondents took courses during their SMPs in biochemistry, cell biology, physiology and neurosciences (data not shown). Given the density and complexity of these advanced courses, an adjustment period is usually required by most students freshly entering medical school. However, SMP-students have the benefit of additional time to develop the necessary study skills that will improve their overall performance as medical students. Another possibility is differences in their pedigree as students. Students who attended an SMP likely had to do so because of their undergraduate careers being inconsistent, ultimately leading to a lower GPA and/or MCAT. It is reasonable to suggest that these students viewed their respective SMPs as an opportunity to re-define themselves as students, and fully commit themselves to academic success. Both of these scenarios highlight the benefits of a rigorous master’s program in acclimating students for the challenges of medical school, thus, strengthening the value of such programs.

Our second key finding is that while more SMP-students tend to engage in research on average, there is no significant difference in the number of research publications between SMP and traditional medical students (Fig 4). These findings are notable given the fact that many SMPs (especially the SMP at UTCOM where the majority of our respondents were enrolled) knowingly integrate and promote research participation into their curriculums. Therefore, it is surprising that SMP students do not have the upper hand when it comes to the number of research publications. This observation could be explained by a number of possibilities; however, no such speculations are verifiable by the collected data. Nevertheless, the data does not support any potential benefit of the master’s program when it comes to research publications, which are highly sought after in residency programs.

This brings us to our third key finding which involves analyzing residency placement differences between traditional and SMP-students. Counter intuitively, despite similar USMLE STEP 1 scores and number of research publications our SMP-respondents placed in competitive residency programs at a significantly (p<0.05) higher percentage than their peers. Our data reveals that SMP-students matched into surgical residency programs at a significantly (p<0.05) higher percentage than their peers (Fig 5). This finding is notable given the lack of statistically significant difference in USMLE STEP 1 scores between the groups and the competitive nature of surgical residencies. This finding may be related to the fact that some medical schools do not have their students retake the courses they completed during their time in the SMP. These SMP-students are therefore granted the opportunity of having multiple months of light coursework during their M1 year. It is certainly possible that these students use their extra time to engage in extracurricular activities and explore their interests in various medical fields. It is easy to see how time spent outside of the library and inside a medical or research setting might lead these students to be better-rounded residency applicants. This finding coupled with the findings regarding USMLE STEP 1 scores more fully establishes the worth and validity of SMP programs.

## Conclusion

In conclusion, our data supports the hypothesis that regardless of their undergraduate credentials, SMP students have the potential to excel in their medical careers, as evidenced by their USMLE STEP 1 performance and their acceptance rates into competitive residencies. We are not suggesting that MCATs be denied their positive-predictive value for success in medical school, we only recommend against the myopic view of looking at all students through the same lens. If given the opportunity, an SMP can redefine a student's ability to succeed in their medical careers.

## Supporting information

S1 File SMP comparative analysis survey. This fifteen-question anonymous survey was designed using Survey Monkey and was subsequently sent out to UT residents and medical students who had taken USMLE STEP 1.
(PDF)
