# Supplementary material for: Premedical special master’s programs increase USMLE STEP1 scores and improve residency placements
Source: PLoS One. 2017 Nov 30;12(11):e0188036. doi: 10.1371/journal.pone.0188036 (PMC5708752; doi:10.1371/journal.pone.0188036)
Supplement: S1 File — This fifteen-question anonymous survey was designed using Survey Monkey and was subsequently sent out to UT residents and medical students who had taken USMLE STEP 1. (PDF) [file pone.0188036.s001.pdf]

Comparative Analysis of Post-Baccalaureate SMP (Masters) Programs for Favorable Outcomes in Medical Careers

1. Did you attend a medical school based in the United States? (If you answer No, please scroll down to the end to complete the survey.)

- ◯ Yes
- ◯ No

2. How old were you when you entered medical school?

- ◯ 21 or younger
- ◯ 22
- ◯ 23
- ◯ 24
- ◯ 25
- ◯ 26
- ◯ 27 or older

3. What was your undergraduate GPA?

- ◯ 2.70-3.00
- ◯ 3.01-3.33
- ◯ 3.34-3.66
- ◯ 3.67-4.00

4. What was your MCAT score (old scale)?

5. What was your STEP 1 score?

6. If applicable, please select the specialty of your residency program.

7. Did you conduct biomedical research during your time in medical school?

- ◯ Yes
- ◯ No

8. If you answered 'Yes' to the above question, how many abstracts/articles did you complete during this time?

- ◯ 0
- ◯ 1
- ◯ 2
- ◯ 3 or more

9. Did you attend a Special Masters Program (SMP) after undergrad? (If you answer No, please scroll down to the end to complete the survey.)

- ◯ Yes
- ◯ No

10. In the space below, please indicate the name of the university you attended for your SMP, as well as the specific name of the program (ex: University of Toledo, Masters of Science in Biomedical Science).

Name of university (ex: University of Toledo)	
Name of program (ex: Masters of Science in Biomedical Science)	

11. What was the length of your SMP Program?

- ◯ 1 Year
- ◯ 2 Years

12. Which of the following courses were offered in your SMP ? Select all that apply.

- [ ] Anatomy
- [ ] Biochemistry/Cell Biology/Genetics
- [ ] Microbiology & Immunology
- [ ] Neuroscience
- [ ] Physiology
- [ ] Pathophysiology
- [ ] Pharmacology
- [ ] Public Health

13. Did you conduct biomedical research during your time in your SMP?

- ○ Yes
- ○ No

14. If so, how many abstracts/articles did you complete during this time?

- ○ 0
- ○ 1
- ○ 2
- ○ 3+

15. How well did the course structure of your SMP improve your performance in that content area on Step 1?

- ○ No effect
- ○ Moderate improvement
- ○ Significant improvement
